# Viral oncogenesis in tumours of the central nervous system: reality or random association? A retrospective study on archived material

**DOI:** 10.1111/jcmm.17064

**Published:** 2022-02-02

**Authors:** Dorel Eugen Arsene, Elena Milanesi, Maria Dobre

**Affiliations:** ^1^ Victor Babes National Institute of Pathology Bucharest Romania; ^2^ National Institute of Neurology and Neurovascular Diseases Bucharest Romania

**Keywords:** glioma, herpesvirus, HPV, meningioma, oncogenesis, polyomavirus

## Abstract

Central nervous system (CNS) tumours have devastating effects and are recurrent, with dismal prognosis (gliomas) or life‐threatening by the compression effect (meningiomas). This disease's aetiology remains debatable. Over the last decade, the hypothesis that human viruses may be implicated in these tumours has been proposed. In this study, our aim is to examine the presence of 11 viruses in the most frequent CNS primary tumours. Using polymerase chain reaction (PCR), we assessed the viral presence in archived, paraffin‐embedded tumour tissues from 114 patients with glioma and meningioma and in the brain tissue from 40 controls lacking tumour pathology. We focused on candidate neuro‐oncogenic types (herpesviridae and polyomaviruses) and on human papillomavirus (HPV). HPV presence, for which involvement in these tumours was hardly investigated, was found to be associated with both tumour categories compared with controls (glioma, *p* = 0.032; meningioma, *p* = 0.032), whereas the presence of the neuro‐oncogenic viruses was found in a negligible number of both categories, suggesting a lack of association with the tumour presence. Moreover, our study reveals a positive correlation between HPV presence and glioma malignancy, and a negative correlation with meningioma grading. Our results suggest that the presence of HPV seems to be significantly associated with primary tumours of the CNS and its meninges.

## 1 BACKGROUND

Central nervous system (CNS) tumours account for a relatively small percentage of human tumours. However, since they cause morbidity and mortality that are disproportionate to their incidence, the mechanisms underpinning their aetiopathogenesis are an important topic of research. There are over 100 different histological types of primary CNS tumours, with an annual age‐adjusted incidence of 28.57 cases per 100,000 inhabitants.^1^

The most common tumours affecting the brain are brain metastases. Among primary tumours, gliomas (comprising glioblastomas, astrocytomas, oligodendrogliomas and ependymomas) are the most prevalent malignant types, while meningiomas are the most common and histologically benign tumours affecting the CNS. Glioblastomas constitute 45% of malignant primary brain tumours and, according to the Central Brain Tumor Registry of the United States data (2008–2012), meningiomas account for 35% of all primary brain tumours.^2^

The aggressiveness of glioblastomas is both histological and clinical, determined by their localization within a rigid space and adjacent to vital areas; for example, patients with glioblastoma, the most aggressive glial primary tumour, have a 5‐year survival rate of 5.1% and a median survival of around 10 months.^3^ Recent approaches for glioblastoma therapy address some pathogenic features and are based on immunological and genetic‐mediated techniques, adding to classical surgery, radio and chemotherapies.^4^, ^5^, ^6^ For meningiomas, standard therapy comprises surgery with or without adjuvant radiation, depending on the tumour grade and the degree of resection.^7^

For any other tumour type, knowing the precise aetiology and pathogenesis would be an essential step to treat, cure or even prevent their onset. Little is known about the aetiology of this brain proliferative condition, and currently, the only established risk factors are ionizing radiation and some rare genetic‐related syndromes.^8^ Moreover, there is growing evidence and consensus regarding the association of certain viruses with brain tumours.^9^ Although some viruses have recently been proposed as therapeutic methods for aggressive glial tumours,^10^, ^11^, ^12^, ^13^ the viral role as an etiological agent is not sufficiently understood.

Overall, approximately 12% of human cancers are supposed to have a viral aetiology.^14^ Human viral oncogenesis is probably characterized by three main features: (a) oncoviruses are necessary but not sufficient for developing cancer; (b) virus‐dependent cancers occur many years after acute infection and after persistent chronic infections; and (c) the immune system can act both towards immunosuppression and chronic inflammation.^15^, ^16^ Viruses activate various intracellular metabolic pathways, with a progressive accumulation of cellular markers that constitute the malignant phenotype. This is achieved by the accumulation of somatic oncogenic alterations (oncogenic strokes) caused by spontaneous mutations or mutations induced by exposure to environmental carcinogens regarding the host's genetic background and the selective pressure imposed by the tissue microenvironment.^17^ The viral oncogenic activity is most recently considered to be oncomodulatory.^18^ Several types of viral infections have been suggested to be potentially associated with CNS tumours, especially glioblastomas. Herpesviridae is a large family of DNA viruses that comprises Herpes Simplex 1 and 2 (HHV1 and HHV2), Varicella zoster virus (HHV‐3), Epstein‐Barr virus (EBV or HHV‐4), Cytomegalovirus (CMV or HHV‐5), Roseolovirus (HHV‐6 and HHV7) and Kaposi's sarcoma‐associated herpesvirus (KSHV or HHV‐8). These neurotropic viruses in the CNS control the activation of glial cells that release pro‐inflammatory mediators and increase oxidative stress, which are associated with neurodegenerative disease pathogenesis. Indeed, the presence of many of these viruses has been related to Alzheimer's disease,^19^ Parkinson's disease^20^ and epilepsy.^21^

Regarding cancer, although EBV and KSHV are officially recognized as carcinogens, other viruses from this family have also been associated with different types of cancer, but their role in carcinogenesis remains unclear.^22^

Emerging evidence has demonstrated the presence of human CMV proteins and nucleic acids in brain tumours in both adults and children,^23^ detecting the virus presence, particularly in patients with high‐grade gliomas. However, data are contrasting and the need for research into the involvement of this virus in gliomas has recently been argued^24^, ^25^ since the CMV presence seems limited only to glioblastoma and not present in other types of glial tumours.^26^, ^27^ Moreover, the real presence of CMV in gliomas is questioned,^28^, ^29^ and single‐cell sequencing does not clearly highlight the presence of this virus^30^

Also, the results regarding the role of the Epstein‐Barr virus (HHV‐4) in glioma genesis are controversial.^31^ Although its presence was detected at higher frequency than other herpesviruses (HHV‐5, HHV‐6 and HHV‐8) in pilocytic astrocytoma, it was not considered responsible for tumorigenesis in those cases.^32^

Also, HHV‐6 is potentially involved in glial oncogenesis. The ability of HHV‐6 to integrate into a chromosomal region that is highly relevant to carcinogenesis, the ability of the HHV‐6 ORF‐1 protein to bind to p53, and its detection of early and late antigens in adult primary and recurrent CNS tumours (more frequently in glial tumours)^33^ make this virus a potential key factor in glioma cases.^34^ However, further studies are considered mandatory.^35^

HHV‐8 or Kaposi's sarcoma‐associated herpesvirus is well known for its oncogenic role in a wide category of haematological malignancies. However, since it also has a strong neurotropism,^36^ it can modulate the proliferation of glioma stem cells.^37^

Moreover, HHV‐2, EBV, and HHV‐6 have been detected in high‐grade glioma tissues, and HHV‐1 and HHV‐2 immunoreactivity have been found in some cases, although these were not detected in their tumour tissue.^38^

Human polyomaviruses represent a diverse group of human pathogens comprising John Cunningham virus (JCV), BK virus and simian virus 40 (SV40), which generally cause asymptomatic infection in healthy individuals. JC and BK viruses are mostly associated with cutaneous, mucosal tumours and bladder carcinoma.^39^ These viruses have been involved in neural oncogenesis, mainly in glioblastoma.^38^ In particular, glioblastoma with a small‐cell neuronal‐like component was mainly related to JCV.^40^ However, the role of this virus in the carcinogenesis of brain tumours remains partially understood.^41^, ^42^

Regarding SV40, most studies on its presence have been performed in the sera of glioma patients without assessing its presence within the brain tissue.^43^ Moreover, differences in the detection methods (viral DNA, mRNA and expression of viral oncoproteins) are considered essential in affirming or denying SV40 involvement as an oncogenic agent.^44^ Finally, a potential involvement of HPV in glioblastoma has been suggested^27^ since this virus has been found to have prognostic implications in glioblastomas.^45^

## 2 METHODS

In this retrospective study, we aimed to detect the presence of various potential oncogenic viruses in the most frequent tumours of the brain and their meninges (glioblastoma and meningioma) compared to patients lacking tumour pathology.

### 2.1 Sample collection

In this study, we enrolled 154 individuals. Fifty‐six patients were diagnosed with meningioma and 58 with glioma (44 glioblastoma, nine grade two diffuse astrocytoma and five anaplastic astrocytoma). Forty patients represented the control group, deceased by other causes and lacking any brain tumour. All study specimens were collected from the Neuropathology Department of the National Institute of Neurology and Neurovascular Diseases. This study was conducted following the guidelines of the Declaration of Helsinki and was approved by the Ethics Committee of the National Institute of Pathology ‘Victor Babes’ (Registration number 53 of 6 December 2017). All the patients or their relatives signed the written informed consent. Only patients with primary tumours of the brain and meninges were enrolled. We excluded metastases (with biology specific to the tissue of origin), glandular (pineal, pituitary) or accessory tissue (eye) tumours.

The first section was stained with haematoxylin‐eosin, and a second histopathological diagnosis was established by an expert pathologist to confirm the initial classification of the cases. All tumours were classified and graded according to the WHO classification of CNS tumours.^46^ Samples with small tissue presenting large necrotic areas or artefacts (ie intraoperative electrical mark) detected by microscopic examination were excluded.

The initial series comprised all primary tumour categories of the brain, including oligodendroglioma, ependymoma and choroid plexus tumours, with a total of 200 cases. However, since most of these other entities were represented by a limited number of cases, we restricted our research to the most common and aggressive brain tumour, grade IV glioblastoma.8963.

The sociodemographic and clinical data of the selected cohorts are reported in Table 1.

**TABLE 1:** Sociodemographic and clinical data of controls and patients involved in the study

	Controls C (*N* = 40)	Meningioma M (*N* = 56)	Glioma G (*N* = 58)		
			Glioblastoma GB Grade IV (*N* = 44)	Astrocytoma A Grade II and III (*N* = 14)	*p‐value*
Sex F (N, %)	19 (47.5%)	36 (64.3%)	15(34.1%)	9(64.3%)	*M vs C p *= 0.101; χ^2^ = 2.687
					*G vs C p *= 0.548; χ^2^ = 0.360
					*GB vs C p *= 0.243; χ^2^ = 1.364
					A vs C *p *= 0.279; χ^2^ = 1.170
Age±SD	65.55 ± 15.63	57.52 ± 12.78	58.27 ± 10.87	47.29 ± 14.28	*M vs C p = 0.007*
					*G vs C p = 0.001*
					*GB vs C p = 0.017*
					*A vs C p *< 0.001
Localization (N)	Cortex=25	Convexity=35	Temporal=16	Frontal=6	
	Striatum=4	Basal=21	Frontal=11	Temporal=4	
	Hippocampus=3		Parieto‐occipital=5	Cerebellum=1	
	Mesencephalon=3		Parietal=3	Fronto‐bilateral=1	
	Hippocampus=3		Temporo‐parietal=3	Fronto‐temporal=1	
	Cerebellum=2		Fronto‐temporal=2	Temporal‐lateral ventricle=1	
	Cingulate gyrus=1		Cerebellum=1		
	Pons=1		Fronto‐parietal=1		
	White matter=1		Temporal‐lateral ventricle=1		
			Third ventricle=1		
Grading		Grade I, benign=38			
		Grade II, atypical=15		Grade II, diffuse astrocytoma=9	
		Grade III, malignant=3		Grade III, anaplastic astrocytoma=5	

### 2.2 DNA isolation, virus detection and HPV genotyping

A number of 5–10 slides (50 μm) were cut from each sample according to the volume. Before DNA isolation, to remove the paraffin, the sections were placed at room temperature in two baths of xylene and absolute ethanol. Genomic DNA from FFPE tissue sections was isolated using a QIAamp DNA FFPE Tissue Kit (Qiagen, Hilden, Germany), following the manufacturer's protocols. The quantity and purity of the DNA were determined using NanoDrop 2000 (Thermo Scientific, DE, USA).

The presence of HHV1 and HHV2, HHV4–5 and BK, HHV6, HHV7, HHV8, JCV and SV40 viruses was detected using dedicated Genesig Advanced kits (Primerdesign Ltd., York House, School Lane, Chandler's Ford, United Kingdom) by qPCR, following the instructions of the manufacturer on the ABI7500 fast (Applied Biosystem, MA, USA). Each kit comprises a positive control that contains templates for the targets in the test and an endogenous control to confirm the extraction of a valid biological template.

The presence of HPV has been detected by PCR using consensus primers targeting the L1 region, GP5+/GP6+ primer set,^47^ as described by Yoshida et al.^48^ This generated a 140‐bp‐long fragment from the HPV L1 structural gene. The amplified DNA was s8998u9ubjected to electrophoresis on 2% agarose gel and then visualized by ultraviolet illumination using ethidium bromide. For all the samples, the DNA integrity was verified by PCR amplification of β‐Globin gene as previously reported.^48^ PCR reactions that were negative after the first round were subjected to a further 30 rounds of PCR amplification using the same primers (auto‐nested PCR).

For samples found HPV positive by auto‐nested PCR, genotyping was performed using the AMPLIQUALITY HPV‐TYPE EXPRESS v3.0 (AB Analitica, Padua, Italy) that allows the identification of the presence of HPV and the detection of 40 HPV genotypes: HPV 6, 11, 16, 18, 26, 31, 33, 35, 39, 40, 42, 43, 44, 45, 51, 52, 53, 54, 55, 56, 58, 59, 61, 62, 64, 66, 67, 68 (68a e 68b), 69, 70, 71, 72, 73, 81, 82, 83, 84, 87, 89, 90. This kit is IVD (In Vitro Diagnostic) marked for detecting HPV in histological FFPE samples. With this method, we did not obtain PCR products after the first amplification. Thus, PCR reactions were subjected to a further 30 rounds of PCR amplification (auto‐nested PCR). In order to validate the specificity of the in‐house PCR, each experiment was performed using positive and negative controls for H39pi9p9piuprupii9PV. The positive control was represented by a DNA isolated from cervix carcinoma and the negative control by NTC (not template control) in order to verify the absence of cross contamination among the analysed samples.

### 2.3 Statistical analysis

Statistical analysis was performed using the Statistical Package for the Social Sciences (SPSS version 17.0). Continuous variable (Age) was tested with the t test. For categorical variables, the frequency, with percentages, was reported and assessed for the two groups by chi‐square test if the number of patients in a subgroup was ≥5, and when the number was less than 5 in any cell, the Fisher exact test was applied.

## 3 RESULTS

A total of 154 individuals were involved in the study, including 79 females (51.3%) and 75 males (48.7%). The mean age of the study population was 58.89 years (SD ±14.01). The participants were grouped into three groups according to the diagnosis of meningioma (*n* = 56), glioma (*n* = 58, including 44 glioblastoma and 14 astrocytoma) and control (*n* = 40). The groups were homogenous for sex but not for age since the control group was older than the experimental group (*p* < 0.05). Regarding grading, in the meningioma group, most of the tumours were of grade I (67.86%), followed by grade II (26.78%), and only 5.36% were of grade III. The glioma group comprised 75.86% glioblastoma, grade IV, and 24.14% of astrocytoma. Among these, 64.28% were diffuse astrocytomas (grade II), and 35.72% were anaplastic astrocytomas (grade III).

HPV was detected in 20.78% of 154 analysed samples, with three positive cases in the control group, 14 in the meningioma group and 15 in the glioma group (13 in the glioblastoma and 2 in the astrocytoma subgroup).

**FIGURE 1:**
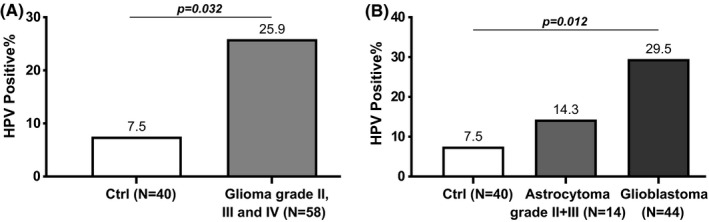
Percentage of positive HPV cases in the control and glioma groups: A, comparison between controls and patients with glioma, B, distribution of HPV‐positive cases in glioma groups stratified in astrocytoma and glioblastoma

**FIGURE 2:**
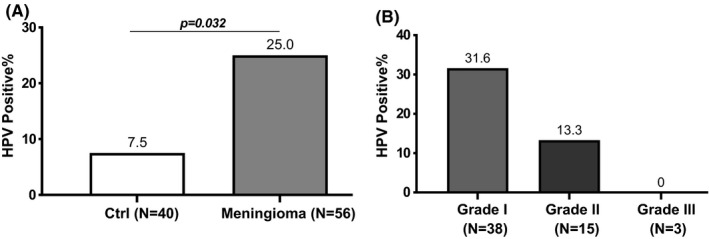
Percentage of positive HPV cases in control and meningioma groups: A, comparison between controls and patients with meningioma, B, distribution of HPV‐positive cases in meningioma group stratified by grading

HPV presence was more frequent in patients with glioma compared to the control group, with a significance of *p* = 0.032 (Figure 1a) and in the glioblastoma subgroup compared with controls (*p* = 0.012) (Figure 1b). No statistical difference was found between the astrocytoma and the control groups. A statistical significance was also observed when comparing the meningioma group with the control group (*p* = 0.032) (Figure 2a). In the meningioma group, the presence of HPV was more frequent in meningioma of grade I, benign (12 positive cases out of 38, 31.6%) than in grade II, atypical (2 positive cases out of 15, 13.3%), and grade III, malignant where no positive cases were detected in the three cases (Figure 2b).

Regarding localization, no statistical difference was observed between convexity meningioma (9 HPV‐positive cases out of 35) and basal meningioma (5 HPV‐positive cases out of 21) (Additional file 1: Table S1).

The presence of HPV in the 32 positive cases was validated using an IVD kit. Specifically, after the first round of PCR with this kit and after the reverse hybridization, in all the analysed samples, we visualized the band of amplification for the endogenous control, demonstrating that the reaction succeeded and the integrity of DNA. After the auto‐nested PCR, we obtained, as expected the band of the endogenous control, and the band for the conserved HPV sequence (universal HPV band), demonstrating the presence of HPV. However, none of the 40 genotypes tested were found in our groups.

An analysis examining the HPV frequency in age categories (age ≤60 and age >60) was conducted in the entire selected cohort and in the three groups. No statistical difference was found between the two age categories in any analysed group (Table 2).

**TABLE 2:** Distribution of HPV‐positive cases in age categories in the different analysed groups

	All individuals (*N* = 154)		Controls (*N* = 40)		Meningioma (*N* = 56)		Glioma (*N* = 58)	
	Age≤60	Age>60	Age≤60	Age>60	Age≤60	Age>60	Age≤60	Age>60
HPV₋	62	60	16	21	21	21	25	18
HPV+	14	18	0	3	8	6	6	9
	χ^2^ = 0.507 *p *= 0.476		Fisher *p *= 0.262		χ^2^ = 0.215 *p *= 0.643		χ^2^ = 1.471 *p *= 0.225	

The distribution of HPV‐positive and HPV‐negative cases in more stringent age categories is reported in (Additional file 1: Table S2).

The presence of HPV in sex categories was also investigated, finding that, in the entire cohort (*N* = 154), the number of HPV‐positive cases was higher in the female group (26.6%) than in the male group (14.7%) but without statistical significance (χ^2^ = 3.318, *p* = 0.069). This trend of difference between males and females was also observed in the control, glioma and meningioma groups, again without statistical significance. (Additional file 1: Table S3).

HHV4‐EBV was identified in 4.5% of total samples (3 positive cases in the control group, 1 in the meningioma group, 1 in the glioblastoma subgroup and 2 in the astrocytoma subgroup). No statistical difference was found regarding the presence of this virus among the groups. One of the positive patients with glioblastoma had a co‐infection with HPV and another with HHV6.

HHV6 was detected in the same percentage as HHV4, but with a different distribution, with four positive cases in the control group, 1 in the meningioma group, and 1 in each glioblastoma and astrocytoma subgroups. Again, no statistical differences among the groups were shown. Of note, the patient with glioblastoma HHV6 positive presented with co‐infection with HPV.

HHV7 was detected only in one patient with meningioma with HPV co‐infection, whereas HHV1–2, HHV5‐CMV, HHV8, JCV, BKV and SV40 were not detected in the control group or in the patient group. All the results are shown in Table 3.

**TABLE 3:** Presence of all the analysed viruses in controls, patients with meningioma and glioma

	Controls C (*N* = 40)	Meningioma M (N = 56)	Glioma G (*N* = 58)		*p‐value*
			Glioblastoma GB (*N* = 44)	Astrocytoma A (*N* = 14)	
HHV1‐2 (N+, %)	0	0	0	0	‐‐
HHV4‐EBV (N+, %)	3 (7.5%)	1 (1.78%)	1(2.3%)	2(14.3%)	*M vs C p *= 0.305
					G vs C *p *= 0.686
					*GB vs C p *= 0.343
					*A vs C p *= 0.595
HHV5‐CMV (N+, %)	0	0	0	0	‐‐
HHV6 (N+, %)	4 (10%)	1 (1.78%)	1(2.3%)	1(7.1%)	*M vs C p *= 0.157
					G vs C *p *= 0.222
					*GB vs C p *= 0.187
					A vs C *p *> 0.999
HHV7 (N+, %)	0	1 (1.78%)	0	0	‐‐
HHV8 (N+, %)	0	0	0	0	‐‐
JCV (N+, %)	0	0	0	0	‐‐
BKV (N+, %)	0	0	0	0	‐‐
SV40 (N+, %)	0	0	0	0	‐‐
HPV (N+, %)	3 (7.5%)	14 (25%)	13(29.5%)	2(14.3%)	*M vs C p = 0.032*
					*G vs C p = 0.032*
					*GB vs C p = 0.012*
					*A vs C p *= 0.595

## 4 DISCUSSION

In this study, we explored the presence of infectious agents belonging to the Herpesviridae family (HHV‐1, HHV‐2, HHV4‐EBV, HHV5‐CMV, HHV6, HHV7 and HHV8), polyomaviruses JCV, BKV, SV40 and HPV in patients with the most common brain tumours (glioma and meningioma) compared to the control group. We found that the most frequent virus in our analysed cohort (40 controls, 56 meningiomas and 58 gliomas) was represented by HPV, with 20.78% of positive cases. Specifically, HPV was detected in 25% of meningioma cases, 25.86% of glioma cases and 7.5% of control samples. Hence, HPV DNA presence was statistically more frequent in patients with glioma and meningioma compared to the controls (*p* = 0.032 and *p* = 0.032, respectively). Of note, HPV positivity in the glioma group was more present in the glioblastoma subgroup (29.5%) compared to the controls (*p* = 0.012).

A striking phenomenon is the negative correlation between HPV positivity and meningioma grading, with the highest frequency of positive cases in grade I meningioma (31.6%), followed by grade II (13.3%), and no cases in grade III (malignant) tumours. This could be due to genetic abnormalities specific to each meningioma group (benign, atypical and malignant), which could interfere with the viral infection. Since the spectrum of histological types of meningeal tumours is remarkably wide, ranging from epithelioid cells to mesenchymal ones (as is the fibrous variant), such particular interactions between the cell of origin (the arachnoid cap cell) and HPV are supposed to generate mostly benign categories of meningothelial proliferation.

No statistical association was observed between the other selected viruses and the brain tumours analysed in our study.

Our results on HPV in glioma and glioblastoma parallel those reported by Vidone and collaborators^45^ and by the research groups of Hashida^27^ and Adnan,^49^ who found 25–28% HPV‐positive cases in patients with glioblastoma, emphasizing the possible role of HPV in the pathophysiological mechanism of this tumour. A higher trend of HPV positivity in glioblastoma cases (39.3%) was recently found by Limam and collaborators.^50^ Because of the positive correlation between HPV presence and glioma malignancy observed in our study (14.3% in grade II and III astrocytoma and 29.5% in glioblastoma), we could speculate that HPV acts as a trigger for malignant transformation of normal glial cells: more cells infected generate a more intense proliferation index, increasing tumour aggressiveness.

Again, in line with our findings, Hashida's study did not show a correlation between CMV and HHV‐8 or EBV presence with glioblastoma.^27^ In our study, HPV DNA detection was performed using two methods (in‐house PCR and a commercial IVD kit). None of the 40 HPV genotypes available in the kit were detected in our positive samples. This could mean that other HPV genotypes are involved in cerebral oncogenesis, or that the IVD kit, designed for detecting HPV in other tissues, such as mucosa or epidermis, is not adequate for processing brain tissue. In both procedures (in‐house PCR and the IVD kit), the virus was detectable only after auto‐nested PCR. This can be due to the small viral HPV DNA copies in brain tissues or tissue processing with large periods of formalin fixation.

Regarding viruses belonging to the herpesviridae family, our results showing a lack of association between CMV and brain tumours parallel what was found by Holdhoff and collaborators,^26^ in other studies, CMV and other herpesviridae, including EBV, have been detected in glioblastoma and other gliomas.^50^, ^51^, ^52^ Of note, some of these studies were performed without control cases, leading to a partial conclusion about the oncogenic role of these viruses in glioma and glioblastoma.^53^ The lack of positivity for HHV‐8 (even in the control tissue) disagrees with other studies, which affirm its positivity in 63.3% of healthy postmortem brain tissue.^36^ In this cohort study, HHV‐6 was more frequently detected in low‐grade glioma (7.1%), almost as in control cases (10%), compared to glioblastoma (2.3%) or meningioma (1.78%). This agrees with other studies that found a higher presence of this HHV type in lower‐grade gliomas.^54^

Herpes viruses, mainly HHV‐6, followed by HHV‐1, HHV‐3, EBV, HHV‐2 and CMV, are present in relatively high percentages in the facial or trigeminal ganglia in the general population,^55^ and their role in oncogenesis has not been established. The analysis of copy number aberrations in astrocytomas of different malignancy grades revealed that the identified genetic regions driving the cancer pathogenesis were interestingly associated, besides inflammation, also with HPV and Herpes simplex infection pathways.^56^ The peripheral presence of such an infection does not mean that the infection propagates to the profound structures of the nervous system. The trajectory of the viral agent could indeed be stopped by the microenvironment conditions of the peripheral ganglia. This mechanism may explain the low frequency of HHV‐positive cases detected in our entire cohort.

Finally, in our cohort, the presence of polyomaviruses (JC, BK or SV40) was not detected. Data from the literature affirm that, in adulthood, a large percentage of the population has been infected with JC and BK viruses. Indeed, a seroprevalence of 39% for JC and 82% for BK was detected in other studies, with the profile of young subjects being similar to adults, demonstrating that primary exposure occurs precociously in life.^57^ However, these data derived from seric determinations and the presence of these polyomaviruses in the brain tissue were not determined in parallel. Hence, the viruses could be latent only in circulating mononuclear blood cells or in the cells of the proximal renal tubule, reactivating in immunocompromised individuals.

## 5 CONCLUSIONS

Overall, our findings, although based on a small number of cases, show a significantly increased frequency of HPV in glioblastoma and meningioma compared to normal brain tissue and suggest the non‐involvement of herpes and polyomaviruses in the investigated brain tumours. However, the detection of a virus within the tumour tissue is not sufficient to affirm its role as a causative agent. Further studies on larger cohorts are needed to clarify the role of these infectious agents in brain tumours and to develop new preventive or therapeutic strategies. Since HPV has been found to have prognostic implications in glioblastomas, and the HPV vaccine in order to prevent cervical cancer cases has established success, a brain tumours prevention through vaccine may be considered.

## CONFLICT OF INTEREST STATEMENT

The authors declare that they have no competing interests.

## AUTHOR CONTRIBUTION

**Dorel Eugen Arsene:** Conceptualization (lead); Resources (lead); Supervision (lead); Writing‐original draft (lead). **Elena Milanesi:** Data curation (lead); Investigation (lead); Methodology (equal); Writing‐review & editing (equal). **Maria Dobre:** Formal analysis (lead); Investigation (lead); Methodology (equal); Writing‐review & editing (equal).

## Supporting information

Supplementary Material

## Data Availability

All data generated or analysed during this study are included in this published article and its additional information files.
