# Supplementary material for: Viral oncogenesis in tumours of the central nervous system: reality or random association? A retrospective study on archived material
Source: J Cell Mol Med. 2022 Feb 2;26(5):1413–20. doi: 10.1111/jcmm.17064 (PMC8899179; doi:10.1111/jcmm.17064)
Supplement: Supplementary file 1 — Supplementary Material [file JCMM-26-1413-s001.docx]

**Additional File 1**

**Viral oncogenesis in tumors of the central nervous system: reality or random association? A retrospective study on archived material**

**Dorel Eugen Arsene ^1,2^, Elena Milanesi *^1^ and Maria Dobre ^1^**

^1^ Victor Babes National Institute of Pathology, 050096 Bucharest, Romania;

^2^ National Institute of Neurology and Neurovascular Diseases, 041914 Bucharest, Romania;

* Corresponding author: [elena.k.milanesi@gmail.com](mailto:elena.k.milanesi@gmail.com)

**Table S1:** Distribution of HPV positive and negative cases in meningiomas according to tumor localization (Convex and Basal)

|  | **HPV** | |  |  |
| --- | --- | --- | --- | --- |
| **Meningioma (N=56)** | **Neg (N, %)** | **Pos (N, %)** | **Significance** | |
| **Convex (N=35)** | 26 (61.9%) | 9 (64.3%) | *Fisher's p= 0.999* | |
| **Basal (N=21)** | 1. (38.1%) | 1. (35.7%) |  |  |

**Table S2:** Distribution of HPV positive and negative cases in different age categories in the entire cohort (N=154) and in control (N=40), meningioma (N=56) and glioma (N=58) groups

|  |  |  |  |  |  |  |
| --- | --- | --- | --- | --- | --- | --- |
|  |  |  |  |  |  |  |
|  |  | **Entire cohort (N=154)** | | | |  |
|  |  | **Age≤45** | **Age 46-60** | **Age 61-70** | **Age>70** |  |
|  | **HPV -** | 23 | 38 | 38 | 23 |  |
|  | **HPV+** | **2** | **12** | **14** | **4** |  |
|  |  |  |  |  |  |  |
|  |  |  |  |  |  |  |
|  |  | **Controls (N=40)** | | | |  |
|  |  | **Age≤45** | **Age 46-60** | **Age 61-70** | **Age>70** |  |
|  | **HPV -** | 3 | 12 | 9 | 13 |  |
|  | **HPV+** | 0 | 0 | **2** | **1** |  |
|  |  |  |  |  |  |  |
|  |  |  |  |  |  |  |
|  |  | **Glioma (N=58)** | | | |  |
|  |  | **Age≤45** | **Age 46-60** | **Age 61-70** | **Age>70** |  |
|  | **HPV -** | 10 | 15 | 14 | 4 |  |
|  | **HPV+** | **1** | **5** | **7** | **2** |  |
|  |  |  |  |  |  |  |
|  |  |  |  |  |  |  |
|  |  | **Meningioma (N=56)** | | | |  |
|  |  | **Age≤45** | **Age 46-60** | **Age 61-70** | **Age>70** |  |
|  | **HPV -** | 10 | 11 | 15 | 6 |  |
|  | **HPV+** | **1** | **7** | **5** | **1** |  |
|  |  |  |  |  |  |  |
|  |  |  |  |  |  |  |
|  |  |  |  |  |  |  |
|  |  |  |  |  |  |  |

**Table S3:** Distribution of HPV positive and negative cases in sex categories in the entire cohort (N=154) and in control (N=40), meningioma (N=56) and glioma (N=58) groups

|  |  |  |  |  |  |  |  |  |
| --- | --- | --- | --- | --- | --- | --- | --- | --- |
|  |  | **Entire cohort (N=154)** | |  |  | **Controls (N=40)** | |  |
|  |  | **M** | **F** |  |  | **M** | **F** |  |
|  | **HPV - (N)** | 64 | 58 |  | **HPV - (N)** | 21 | 16 |  |
|  | **HPV+ (N, %)** | **11 (14.7%)** | **21 (26.6%)** |  | **HPV+ (N, %)** | **0 (0%)** | **3 (15.8%)** |  |
|  |  | χ^2^=3.318, p=0.069 | |  |  | Fisher p=0.098 | |  |
|  |  |  |  |  |  |  |  |  |
|  |  |  |  |  |  |  |  |  |
|  |  |  |  |  |  |  |  |  |
|  |  | **Meningioma (N=56)** | |  |  | **Glioma (N=58)** | |  |
|  |  | **M** | **F** |  |  | **M** | **F** |  |
|  | **HPV - (N)** | 17 | 25 |  | **HPV - (N)** | 26 | 17 |  |
|  | **HPV+ (N, %)** | **3 (15%)** | **11 (30.5%)** |  | **HPV+ (N, %)** | **8 (23.5%)** | **7 (29.2%)** |  |
|  |  | Fisher p=0.334 | |  |  | χ^2^=0.233, p=0.629 | |  |
|  |  |  |  |  |  |  |  |  |
|  |  |  |  |  |  |  |  |  |
|  |  |  |  |  |  |  |  |  |
